# Data we can trust

**DOI:** 10.1172/JCI162884

**Published:** 2022-08-01

**Authors:** Sarah Jackson, Corinne L. Williams, Kathleen L. Collins, Elizabeth M. McNally

## Abstract

“On behalf of all authors of the submission, I warrant that the work is original and scientifically accurate ...” If you’ve submitted a manuscript to the *Journal of Clinical Investigation* or *JCI Insight*, this phrase should sound familiar. This statement is the very first thing that we ask authors to verify for every new submission. While this may seem like a simple formality or just another screen to click through, certifying the accuracy of information presented to the journal is essential to the publishing process and scientific integrity. Data accuracy forms the foundation of the scientific enterprise, and without it, the enterprise risks crumbling.

To a great degree, scientific investigation and publishing are built upon trust in authors. But in representing the American Society for Clinical Investigation as Editors, we employ safeguards to help verify the quality of work in the *JCI* Family of Journals. The first pillar is the Editorial Board of academic and professional editors. With broad scientific knowledge, our Editorial Boards scrutinize the rigor and quality of manuscripts. We view this evaluation as a hallmark of our journals and reflection of our professional integrity. The second pillar of quality is peer review. For decades, scientific publishing has relied on input provided by subject matter experts who provide technical analyses of the research under consideration as well as an assessment of the work’s importance. We simply could not adequately assess the varied research that we publish without the contribution of our reviewers. A third pillar instituted by the *JCI* family of journals now involves an artificial intelligence–based (AI-based) screen for overt data issues.

In July 2021, the *JCI* and *JCI Insight* implemented additional checks using Proofig (https://www.proofig.com/), a software that evaluates images for duplicated content. We apply this screen to each manuscript prior to full acceptance. For those of you who carefully read the terms of your submission agreement, this is disclosed in point 3. Prior to adopting Proofig, our professional Editors laboriously screened blots and images, as we described in a previous editorial (1). This prior experience, which relied on manual scrutiny of manuscripts, made it evident that there were a host of image issues being missed in peer review and by our Editorial Board. Activity on PubPeer, a website that catalogs data concerns in post-publication manuscripts, supports the concept that data integrity issues are present across many, if not all, biomedical journals. In 2019, we reported that our manual screening process resulted in rejection of 1% of submissions to the *JCI* that had otherwise satisfied the peer and Editorial Board review processes. After a year of AI-based detection of image duplication, that number has tripled (3%), resulting in 13 rejections at the *JCI*. Similarly, *JCI Insight* has rejected 7 manuscripts in the last year due to issues discovered through image duplication screening. There were an additional 3 manuscripts withdrawn from consideration from both journals after authors were queried about image duplications and 2 more papers rejected due to our manual screening of Western blot data. It is also noteworthy that only one of these manuscripts had an image issue identified by a reviewer.

What does it mean that we are increasingly detecting inappropriate image duplication? First, the software is obviously better at identifying duplications in some types of data than our professional Editors were. Bear in mind that the manual screening process had already proven to generally identify more issues than peer review did. Is this a sign that cheating runs rampant in modern science? No doubt the increasing difficulty in securing jobs and grants presents an enormous potential pressure for individual investigators. But might some of the problems be less sinister? The size and scope of the average manuscript has grown, and it is not uncommon for an article to have 8–10 main figures, with 10–20 supplemental figures, and 10 or more coauthors covering a broad range of technical expertise. To err is human — is it any wonder that mistakes will be made? In that regard, we hope that the new NIH policies on data sharing taking effect in 2023 will help authors to avoid simple mistakes involving data organization (2). The NIH will require funded investigators to articulate prospectively how data will be archived and shared. For some types of data, public repositories have provided a vehicle for easy data access and sharing, but for many data sets, no such repositories exist. Individual institutions will likely also need to provide investigators with support and resources to preserve and share data. With this, we hope there will be more guidance on best practices for data acquisition, file naming conventions, storage, and transfer. More rigorous oversight of archiving could mitigate the propensity for error that accompanies many steps in data management for complex manuscripts.

We are not naive, and we understand that some forms of malevolent data fabrication may be undetectable. This type of active and knowing fraud is hopefully uncovered when the discoveries cannot be replicated. Yet we find ourselves routinely identifying problematic images in submissions, such as the use of the same image to represent distinct samples or treatments, and we must arbitrate the author’s intent behind these errors. We rely on a standard evaluation process, allowing authors an opportunity to review and explain how images came to be duplicated. But as stewards of the *JCI* and *JCI Insight*, the Editors always reserve the right to reject a manuscript for data inaccuracies. Often, we cannot make a clear determination as to whether the error is fraud or simple carelessness. At the end of the day, an inability to accurately present the data erodes our confidence in all the manuscript’s content. If we can spot obvious mistakes, our trust in other aspects of the manuscript can no longer be an inherent assumption. We hope each author takes every measure to ensure data accuracy and foster scientific integrity. With this article, we implore every author to redouble their efforts. Your good name, and ours, depend on data we can trust.

## Version 1 08/01/2022

Electronic publication
